# College and Hospital Notes

**Published:** 1910-08

**Authors:** 


### COLLEGE AND HOSPITAL NOTES

Dr. Charles R. Borzilleri, who established the Columbus Hospital in Buffalo about two years ago, announces that an increase to twice its present size will be made soon. The following staff of the hospital is announced also: consulting—internal medicine, Henry C. Buswell; surgery, Marshall Clinton; gynecology, Earl P. Lothrop; nose and throat, Max Kaiser and James J. Mooney; nervous diseases, James Wright Putnam; ophthalmology, F. Park Lewis; orthopedics, B. Bartow; obstetrics, P. W. Van Peyma; genitourinary diseases, Nelson W. Wilson; pediatrics, I. W. Snow; dermatology, Grover W. Wende; pathology, Norman K. MacLeod; radiographer, A. W. Bayliss. Attending—internal medicine, Thomas J. Walsh, Horace L. Grasso, and A. J. Getola; surgery and gynecology, Charles R. Borzilleri; Charles Battaglia, and Edward E. Haley; nose and throat, Gianfrancisco; nervous diseases, F. Strozzi; orthopedics, Prescott Le Breton; obstetrics, L. Franklin Anderson; genitourinary, Joseph J. Giambrone; pediatrics, John A. Ragone and Jacob M. Bayliss; dermatology, Grover W. Wende; pathology, Frank A. Valanti; anesthetist, L. Franklin Anderson; dental surgery, J. Pauzica.

